# Preliminary Characterization of Bulgarian Forest Honeys: Oak Honeydew and Coniferous Varieties

**DOI:** 10.3390/foods14244298

**Published:** 2025-12-14

**Authors:** Elisaveta Mladenova, Ralitsa Balkanska, Rositsa Shumkova

**Affiliations:** 1 Faculty of Chemistry and Pharmacy, Sofia University, James Bourchier Boulevard, 1, 1164 Sofia, Bulgaria; 2 Department “Special Branches”, Institute of Animal Science, Kostinbrod, Agricultural Academy, 1113 Sofia, Bulgaria; r.balkanska@ias.bg; 3 Research Centre of Stockbreeding and Agriculture, Agricultural Academy, 4700 Smolyan, Bulgaria; rositsa6z@abv.bg

**Keywords:** oak honeydew honey (OHH), coniferous honeydew honey (CHH), physicochemical properties, quality parameters, elemental profile

## Abstract

The objective of this work was to determine and compare a comprehensive set of quality markers, including main physicochemical properties and element profiles, in samples of Bulgarian oak honeydew honey (OHH) and coniferous honeydew honey (CHH). This investigation utilized a total of seventeen honey samples from Bulgaria harvested in 2022. The sample set comprised ten oak honeydew honey samples, sourced from the Burgas region, and seven coniferous honeydew honey samples obtained from the Smolyan region. The parameters of OHH samples varied within the following ranges: color (80–134 mm Pfund), water content (15.20–18.40%), electrical conductivity (0.80–1.33 mS/cm), specific optical rotation (2.25–12.50 ${\left[\alpha \right]}_{D}^{20}$), pH (3.92–4.50), total acidity (29.80–36.80 meq/kg), diastase activity (18.36–27.58 Gothe units), invertase activity (56–196 U/kg), proline content (155–477 mg/kg), and hydroxymethylfurfural (3.28–8.94 mg/kg). The CHH samples gave the following results: color (40–87 mm Pfund), water content (16.40–19.00%), electrical conductivity (0.80–1.26 mS/cm), specific optical rotation (−17.50–(−11.50 ${\left[\alpha \right]}_{D}^{20}$)), pH (3.40–3.75), total acidity (25.80–39.40 meq/kg), diastase activity (23.15–26.05 Gothe units), invertase activity (69–138 U/kg), proline content (287–651 mg/kg), and hydroxymethylfurfural (1.50–3.96 mg/kg). The elements Ca, Cu, Fe, Mg, and Mn were determined by Flame Atomic Absorption Spectrometer (FAAS), while Flame Atomic Emission Spectrometry (FAES) was used for K and Na determination. Inductively Coupled Plasma Optical Emission Spectrometry (ICP-OES) analysis was used to assess six elements (Al, Ba, Co, P, Sr, Zn). The elements Cd, Ni, and Pb were determined by Electrothermal Atomic Absorption Spectrometry (ETAAS). Potassium is the most abundant macro element in all investigated samples ranging 2332–2370 µg/g in CHH and 1846–1878 µg/g in OHH. Other examined elements are in the following descending order, Mg > P > Na > Ca > Mn > Al > Fe > Zn > Cu > Ba > Sr, presenting in µg/g levels, while Pb > Ni > Co > Cd are present in µg/kg levels. This work constitutes the first report on the physicochemical parameters and chemical elements of coniferous honeydew honey from Bulgaria.

## 1. Introduction

Honey is a widely appreciated natural product. It has nutritional and pharmaceutical benefits and it has been used in traditional medicine for centuries [1,2]. Honeybees collect nectar, pollen, and honeydew. After that, they mix them with digestive enzymes and store the received products in the honeycomb cell [3,4]. Nectar and pollen are obtained from flowers and honeydew is derived from plant-sucking insects [5]. Honeydew honey is produced by bees from secretions of living parts of plants or excretions of plant-sucking insects (mainly aphids from *Hemiptera* order) of the plants [6]. There are many species from *Hemiptera* which produce sugar-containing secretions from the phloem sap of different plants. These honeydew droplets are collected by honeybees and used to make honeydew honey [7,8]. It can be made from leafy or coniferous honeydew [9]. The plants used for honeydew honey production are coniferous trees such as fir (*Abies alba*), spruce (*Picea abies*), but also leafy (deciduous, latifoliae) trees, such as oak (*Quercus* spp.) and lime (*Tilia* spp.) [6,10]. According to the source of the honey, it is classified as blossom honey or honeydew honey [11]. In general, honeydew honey is characterized by a darker color and higher values of electrical conductivity, pH, acidity, and mineral content than nectar honey [12]. Pollen analysis of honey samples and the determination of physicochemical parameters could prevent commercial fraud [13]. Furthermore, differentiation between nectar and honeydew honey is needed to meet consumer demands. In general, it cannot be said which honey is more valued and preferred in the world [6]. Nevertheless, the developing market for honeydew honeys in Europe is leading to increasing demands from consumers and the honey industry for the characterization of these honey types [14]. In general, honeydew production is a dynamic process each year. It depends on the environmental conditions, host plant physiology and phenology, and the developmental biology of the sap-sucking insects responsible for honey production [15].

The production of coniferous and oak honeydew honeys is therefore contingent upon the preservation and stability of their insect populations, highlighting the direct link between apicultural productivity, biodiversity conservation, and the ecological integrity of Bulgaria’s native oak and coniferous forests.

This work is a contribution to the limited knowledge of honeydew honeys in Bulgaria. Oak honeydew honey (OHH) is generally associated with a dark color and the main production of this honey type comes from the Strandzha Mountain (southeastern Bulgaria) where *Quercus* spp. disseminated [16,17]. The OHH is characterized by a unique physicochemical profile, including high mineral content, specific oligosaccharide and polyphenol fingerprint, and significant bioactive properties. It is recognized as a functional food [18].

Mineral elements are minor constituents of honey. They are important for determining its quality [19]. Many studies have also been conducted on the mineral content of honeydew honeys: Jara-Palacios et al. analyzed Spanish honeys [14], Šedík et al. analyzed Slovakian honeys [20], Mara et al. analyzed Italian honeys [21], and Atanassova et al. analyzed Bulgarian honeys [17].

However, various honeydew honeys have been less studied than other types of nectar honeys. Due to the scarce number of papers related to the characterization of oak and coniferous honeydew honeys, a comprehensive analysis of these honey types has been performed.

In fact, the oak and coniferous types are the only honeydew honeys which are produced in the territory of Bulgaria. Therefore, the objective of this work was to determine and compare a comprehensive set of quality markers, including main physicochemical properties and element profiles, in samples of Bulgarian oak honeydew honey and coniferous honeydew honey.

## 2. Materials and Methods

### 2.1. Samples

**Figure 1:**
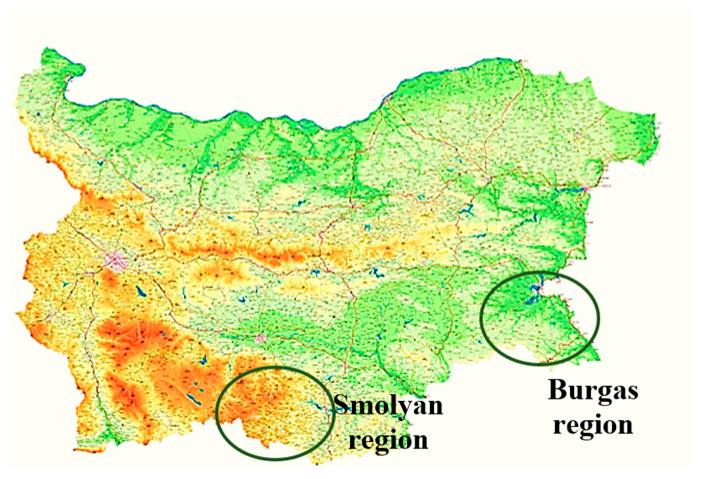
Geographical locations of honey sample collection areas.

This investigation utilized a total of seventeen honey samples from Bulgaria harvested in 2022. The sample set comprised ten oak honeydew honey samples, sourced from the following distinct geographical locations within the Burgas region: Ahtopol (two samples), Sinemorets (three samples), Strandzha Mountain (one sample), Malko Tarnovo (two samples), and Tsarevo (two samples). Additionally, seven polyfloral honey samples with a confirmed admixture of coniferous honeydew honey were included, collected from the Smolyan region (specifically, the Pamporovo resort—one sample, the Smolyan Lakes area—two samples, Momchilovtsi village—one sample, and Zlatograd—three samples). Sample regions are presented in Figure 1. All samples were stored in glass jars for 4 weeks in the dark at 18 °C prior to analysis.

Coniferous honeydew honey is a product of forest beekeeping in the Smolyan region (Southern-central Bulgaria, located in the Rhodope Mountains, see Figure 1). This honey has a brighter color compared to the oak honeydew honey. Coniferous honeydew honey is produced in an ecologically clean region which is rich in coniferous forests. Local beekeepers who collect this honey in the traditional way know that very often this honey can be mixed with polyfloral honey from meadow plants. They know how to choose the location of the apiary in view of the amount of honeydew available. The insufficient presence of significant floral resources often drives bees to forage on this abundant, sugar-rich resource, resulting in a honey with a markedly different biochemical signature. Coniferous honey is very often categorized as forest honey with its increased electrical conductivity value.

All the honey samples were analyzed after confirmation of the honey variety. The pollen analysis was carried out by Bulgarian State Standard for Bee Honey 3050-80 [22]. In brief, ten grams of honey sample are dissolved in 30 mL of distilled water. The solution is centrifuged for 10 min at 10,000 rpm. A drop of the precipitate is transferred to a glass slide. For each honey sample, at least 200 pollen grains were counted.

The samples are labeled on the basis of the values of electrical conductivity. There is no accepted specific quality criterion for different types of honeydew honeys.

### 2.2. Determination of Physicochemical Parameters

A comprehensive analysis of key physicochemical parameters was performed in accordance with the standard methods prescribed by the International Honey Commission [23]. The analyzed parameters included water content, pH, total acidity, electrical conductivity, diastase activity, invertase activity, proline content, and hydroxymethylfurfural (HMF) concentration. Furthermore, the specific optical rotation was determined. Honey color was quantified using a colorimetric approach, with measurements expressed in millimeters on the Pfund scale, facilitated by a Lovibond HoneyColorpod colorimeter [23]. All samples were homogenized before each analytical procedure and three replicates were analyzed.

### 2.3. Determination of Chemical Elements

#### 2.3.1. Sample Preparation Procedure

The digestion procedure of honey samples was performed as described previously by Voyslavov et al. [24]. Amounts of 1.0000 ± 0.0002 g of each sample were weighed with an accurate mass on an analytical balance (Kern ACS 220-4, d = 0.1 mg) in PTFE vessels. A mixture of conc. HNO_3_ (≥69.0% TraceSELECT, Honeywell, Fluka, Seelze, Germany) and 30% H_2_O_2_ (TraceSELECT, Honeywell, Fluka, Seelze, Germany) was added and vessels were put in a microwave digestion system (Ethos Easy, Milestone, Italy). After digestion, samples were quantitatively transferred into centrifuge tubes and filled up to an accurate mass of 15 g (on an analytical balance) using Milli-Q water (Millipore purification system Synergy, Darmstadt, Germany). Before instrumental measurement, the resulting solutions were diluted tenfold with Milli-Q water. Three replicates were developed from each sample. Blank sample was passed through the whole procedure.

#### 2.3.2. Apparatus

Quantitative determination of Ca, Cu, Fe, Mg, Mn, was performed using Flame Atomic Absorption Spectrometer (FAAS, Perkin Elmer AAnalyst 400, Waltham, MA, USA) in the air-acetylene flame mode, at a wavelength and slit width according to the manufacturer’s recommendations and a measurement height in the flame of 7 mm. Elements K and Na were determined in emission mode. Single element standard solutions of each of element (Merck, Darmstadt, Germany) with an initial concentration of 1000 mg/L were used for calibration after appropriate dilution.

The elements Al, Ba, Co, P, Sr, Zn were quantitatively determined by Inductively Coupled Plasma Optical Emission Spectrometry (ICP-OES) on a Jobin Yvon spectrometer (Ultima 2, Jobin Yvon Horiba, Edison, NJ, USA), equipped with a cyclone injection chamber and a concentric atomizer. The plasma power was 1100 W with 12 L/min plasma, 1 L/min carrier, 0.2 L/min auxiliary, and 0.3 L/min cooling argon flow. The measurements were carried out at a torch height of 15 mm. The integration time was 1 s. The wavelengths used were as follows: Al 396.153 nm; Ba 455.403 nm; Co 228.615 nm; P 177.495 nm; Sr 421.552 nm; Zn 213.856 nm. Stock 1000 mg/L single element standard solutions of P (Trace-CERT, Supelco, Bellefonte, PA, USA) and stock multi-element standard solution of 1000 mg/L (ICP Multi-Element Standard Solution IV, CertiPUR, Supelco, Bellefonte, Pennsylvania, United States) for the rest of the elements were used for the preparation of working standard solutions for calibration.

The amount of the elements Cd, Ni, Pb was determined by Electrotermal Atomic Absorption Spectrometry (ETAAS, Perkin Elmer AAnalyst 400 equipped with HGA 900, Waltham, MA, USA). The light source was a hollow cathode lamp. The spectral gap width and wavelengths used were as prescribed by the manufacturer. A volume of 20 µL of the sample solutions was injected. The peak area was used to quantify the concentrations of the elements. Table S1 presents the temperature programs used. Stock multi-element standard solution of 5 mg/L Cd, 50 mg/L Ni, 100 mg/L Pb (GF AAS Multielement standard XVIII, CERTIPUR, Merck KGaA, Darmstadt, Germany) was used for the preparation of working standard solutions for calibration.

Regarding the working range of the elements, three to five standard solutions were prepared for each quantified element. All standard solutions were prepared with Milli-Q water. For stabilization of the standard solutions, suprapure nitric acid (65%, Supelco, Bellefonte, PA, USA) was used in order to obtain 1 mol/L acidified solution.

### 2.4. Statistical Analysis

All analytical measurements were performed in triplicate, and data are presented as mean ± standard deviation. Statistical analysis was performed using SPSS software (Version 23.0 for Windows, IBM Corp., USA). The significance of differences between the mean values of the two honey groups (oak honeydew honey and coniferous honeydew honey) was assessed using a Student’s t-test for independent samples. A probability value of *p* < 0.05 was considered statistically significant. To evaluate the relationships between the various physicochemical parameters and elemental concentrations within the entire dataset, Pearson’s correlation coefficient (r) was calculated. Correlations were considered statistically significant at *p* < 0.05.

## 3. Results and Discussion

In this study, the main pollen types of OHH and CHH are of particular interest. For this reason, only the plant families are presented in Table 1. Combinations of physicochemical parameters and element profiles are used in the characterization of these honey types. In all honey samples were found honeydew elements such as algae and fungal mycelia.

### 3.1. Physicochemical Characterization

The results of the physicochemical analysis for all honey samples are presented in Table 2.

#### 3.1.1. Color

Darker colored honeys such as honeydew honey have a higher mineral content, bioactive compound, and antioxidant capacity than lighter colored honeys [25]. All oak honeydew honey samples have a dark to very dark color (80–134 mm Pfund). In the present study, significant differences were found between the colors of oak and coniferous honeydew honeys. Manzanares et al. gave a range of 71–150 mm Pfund for honeydew honey [26]. Vanhanen et al. obtain similar results for the same parameter [27]. Coniferous honey is characterized by a lighter color compared to oak honeydew honey (Table 2). In a recent study, Tomczyk et al. reported higher values for coniferous honey compared to our results [10]. Kus et al. reported a dark color for Polish fir honeydew honey [28]. Rybak-Chmielewska et al. present high values for color in honeydew honey (*Abies alba*) [29].

The dark color of honeydew honeys is strongly correlated with their high mineral content (as reflected in the electrical conductivity) and with the presence of polyphenols. According to Fernandez-Torres et al., darker colored honeys, such as honeydew honey, have a higher mineral content [30], as in the present study, while this is contrary to the study of Vanhanen et al. for New Zealand honey [27].

It is known that the honey type largely determines its physicochemical properties, as also the color. Physicochemical parameters such as pH, acidity, mineral content, color, and electrical conductivity have been considered as the main characteristics for the differentiation of nectar honey and honeydew honey.

#### 3.1.2. Water Content

Water is the second largest component of honey and is linked to its ripening. Also, it affects honey quality and is related to the condition in the beehive. According to the results presented in this study, the honey samples are characterized by a relatively low water content. The water content of all examined honey samples is below 20%; this indicates that there are no fermentation processes, and that the honey is ripe. Water content is within the international parameters recommended for honey by the European Commission [31]. The average values of water content of the studied honey types are shown in Table 2. Our results for water content in both honey types are similar to those reported by Seijo et al. [15] and Rybak-Chmielewska et al. [29]. The absence of a significant difference suggests that the water content in these honey types is more influenced by apicultural practices and seasonal conditions during the harvest period than by the specific botanical origin of the honeydew. The consistent low water content across both groups confirms their overall high quality and good shelf-life potential, as low water activity effectively inhibits yeast fermentation and spoilage.

#### 3.1.3. Electrical Conductivity

The most diagnostic parameter for distinguishing honeydew honeys from floral honeys is electrical conductivity. It is one of the most important characteristics of honey. According to the Council Directive [15], the electrical conductivity of honeydew honey should be higher than 0.8 mS/cm. Coniferous honeydew honey is also characterized by high values of electrical conductivity (0.92 ± 0.17 mS/cm) compared to nectar honey (not more than 0.8 mS/cm). These results are similar to those found by Rybak-Chmielewska et al. for honeydew honey, mainly from *Abies alba* [29]. In the analyzed oak honeydew honeys, the average value of electrical conductivity ranged from 0.85 to 1.17 mS/cm. This clearly indicates that the honeys can be classified as honeydew. Not significant differences were found between the mean values of electrical conductivity of both honey types. The mean value for OHH (1.01 ± 0.16 mS/cm) unequivocally confirms its strong honeydew character. In contrast, the mean value for CHH (0.92 ± 0.17 mS/cm) was marginally above this limit, indicating a significant but less dominant honeydew component mixed by floral nectars. This pronounced difference can be a direct reflection of the higher mineral content and organic acid content in the phloem sap of oaks and its subsequent concentration through the insect vector and bee processing. The electrical conductivity value is thus a reliable, rapid indicator of the purity and intensity of the oak honeydew source.

#### 3.1.4. Specific Optical Rotation

As general criteria, nectar honey is levorotatory and honeydew honey (or adulterated nectar honey) is usually dextrorotatory [32]. The specific optical rotation of honey is dependent on the ratio between carbohydrates that rotate polarized light to the right (dextrorotatory) and those that rotate polarized light to the left (levorotatory). Fructose turns the plane of polarized light to the left. Glucose, disaccharides, and tri-saccharides rotate polarized light to the right. The higher content of fructose in nectar honey turns the light to the left and the optical activity is negative [33]. If the former group is in the majority, honey will exhibit a positive specific optical rotation. The specific optical rotation of all honeydew honey samples analyzed was positive, in accordance with previous studies of Bulgarian honeydew honey [34] and in agreement with the findings of other authors [35].

The specific optical rotation was levorotatory (negative) for coniferous honeydew honey (Table 2). A negative optical rotation usually can indicate a higher concentration of the levorotatory sugars such as fructose presented in CHH. Gerginova et al. studied three coniferous honeydew samples. The authors present positive and negatives values for these samples. Furthermore, the authors explain that the reasons for the differences in the optical rotation could be the different types of sucking insects and plant species [36]. Primorac et al. compared Croatian and Macedonian honeydew honey samples and also reported positive and negative optical rotations [37]. This parameter (specific optical rotation) is highly influenced by the specific sugar composition. The strong positive rotation in OHH is characteristic of a high content of oligosaccharides such as melezitose, erlose, and raffinose, which are typical of oak honeydew. This finding provides indirect but strong evidence for a fundamentally different carbohydrate profile.

#### 3.1.5. Total Acidity and pH

The total acidity is identical in OHH and CHH samples. Thus, acidity is attributed to a greater presence of organic acids (e.g., gluconic, acetic, formic) [12]. Correspondingly, the pH of OHH was significantly higher (4.26 ± 0.20) than that of CHH (3.59 ± 0.13). On the other hand, there are reports giving a much higher pH value for coniferous honeydew honey from Poland [29,38]. The high acidity with a higher pH is not uncommon in oak honeydew honeys and can be explained by their stronger buffering capacity. The pH of honey can act as a limiting and inhibitory factor for microbial growth. The low pH of honey is also attributed to the presence of hydrogen peroxide, which is produced by the enzyme glucose oxidase. Hydrogen peroxide is produced when glucose oxidase oxidizes glucose to gluconic acid and hydrogen peroxide. This strong antimicrobial agent inhibits the growth of microorganisms. While pH is an important quality parameter, there is currently no legislation that establishes acceptable limits for it [25].

#### 3.1.6. Enzyme Activity (Diastase and Invertase)

The average values for diastase and invertase activity did not differ significantly in OHH and CHH (Table 2). Observed ranges were 19.4–26.0 Gothe units and 78–164 U/kg for oak honeydew honey, compared to 23.86–25.90 Gothe units and 72–128 U/kg for coniferous honeydew honey. These enzymes are primarily of bee origin and are introduced during the ripening process of honey. Their higher activity could be indicative of a higher bee enzyme contribution during the processing of the more complex sugars found in both honey types. Furthermore, enzyme activity is a key marker of honey freshness and minimal thermal or oxidative degradation. Similar enzyme activities in both honey types indicate that their differences are primarily due to mineral content and optical rotation—parameters influenced by plant phloem sap—rather than bee-derived enzymatic processing. The high, similar levels of diastase and invertase uniformly attest to the excellent quality, freshness, and careful handling of both the OHH and CHH samples studied.

#### 3.1.7. Proline Content and Ripeness

The proline content was significantly higher in CHH (468 mg/kg) than in OHH (287 mg/kg) for the samples presented in this paper. Rybak-Chmielewska et al. found higher average proline values for coniferous honeydew honey from Poland [29]. Proline is the major amino acid in honey, derived primarily from bee salivary secretions. It serves as a marker of honey ripeness and, to some extent, authenticity, as it indicates sufficient processing by bees and the absence of artificial sugar syrups. The higher level in CHH may reflect a greater bee effort required to process and ripen the complex honeydew material.

#### 3.1.8. Hydroxymethylfurfural (HMF) and Freshness

The average values of hydroxymethylfurfural (HMF) are 6.28 ± 2.08 mg/kg for oak honeydew honey (OHH) and 2.83 ± 0.87 mg/kg for coniferous honeydew honey (CHH). The mean values are significantly different. Regarding the European legislation, the maximum permissible content of HMF should be lower than 40 mg/kg [31]. Hydroxymethylfurfural is a compound formed from the degradation of fructose in acidic environments and is a sensitive marker of heat exposure and storage time. The low HMF in all samples confirms overall good freshness and a lack of excessive heating.

### 3.2. Elemental Composition

In the resent years, the classification of unifloral honeys from different origins according to their elemental content has gained attention with analytical techniques proving to be powerful tools for botanical and geographical authentication [24]. In our investigation, elements Al, Ba, Ca, Cd, Co, Cu, Fe, K, Mg, Mn, Na, Ni, P, Pb, Sr, and Zn are selected to be quantified in analyzed samples of OHH and CHH. The selected analytes represent essential elements, potential toxic elements, and trace elements as constituents of honeydew honeys. The present study uses two different units for elemental concentrations: µg/g for major and trace minerals (e.g., K, Mg, Ca) and µg/kg for toxic elements (e.g., Cd, Ni, Pb, Co). This distinction is needed because macro elements occur at higher concentrations (hundreds or thousands of µg per gram), while contaminants are present at very low levels (a few µg per kilogram).

Our analysis revealed significantly higher concentrations (*p* < 0.05) of the macro elements Mg, P, Ca, Na, and Mn in OHH compared to the CHH. It is well known that higher element content leads to higher electrical conductivity and darker color of honey [39]. Samples of Bulgarian OHH contain higher levels of all of the studied elements (except Al and K). This is in agreement with the obtained higher values for electrical conductivity and color of OHH compared to CHH (Table 2).

**Figure 2:**
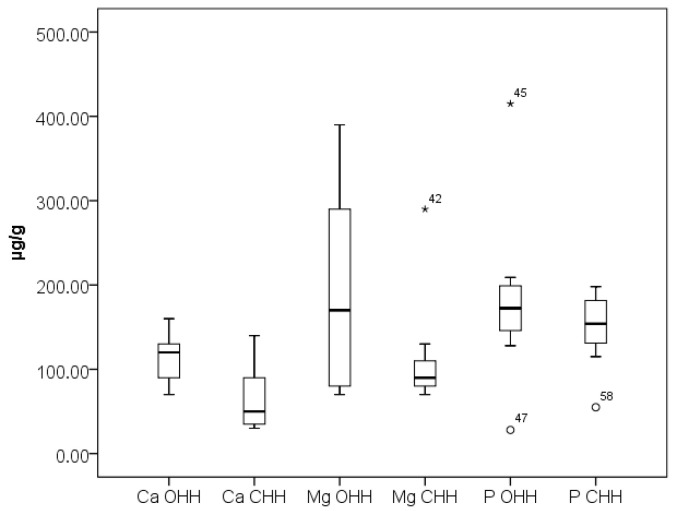
Box plot diagram of contents of Ca, Mg, and P in Bulgarian OHH (oak honeydew honey) and CHH (coniferous honeydew honey) expressed as µg/g. Minimal, maximal, and median values are presented. Legend of outliers: CHH 42 (Mg)—Smolyan Lakes 1 (290 µg/g), OHH 45 (P)—Tsarevo 1 (415 µg/g), OHH 47 (P)—Sinemorets 2 (28 µg/g), CHH 58 (P)—Pamporovo resort 1 (55 µg/g).

Potassium (K) is the most abundant macro element in all investigated samples. The content of K is higher in CHH (2351 ± 19 µg/g) compared to OHH (1862 ± 16 µg/g). Many studies have reported that K is the main mineral, with its values being highly variable [10,13,40]. The concentrations of elements Ca, Fe, K, Mg, Mn, and Sr can be influenced by the composition of the soil and the specific phloem sap upon which the sap-sucking insects feed [41]. Results obtained for the Ca, Mg, and P content in the analyzed OHH and CHH samples are presented in Figure 2. Magnesium (Mg) is the second most prevalent mineral, with a mean value of 191 ± 10 µg/g in OHH and 120 ± 9 µg/g in CHH, followed by P and Ca. This is in agreement with the research of Strecka et al., which reports 67 ± 1 µg/g Mg in oak honeydew honey and 139 ± 2 µg/g Mg in coniferous honeydew honey, and 71 ± 1 µg/g Ca in oak honeydew honey and 94 ± 1 µg/g Ca in coniferous honeydew honey [42].

**Figure 3:**
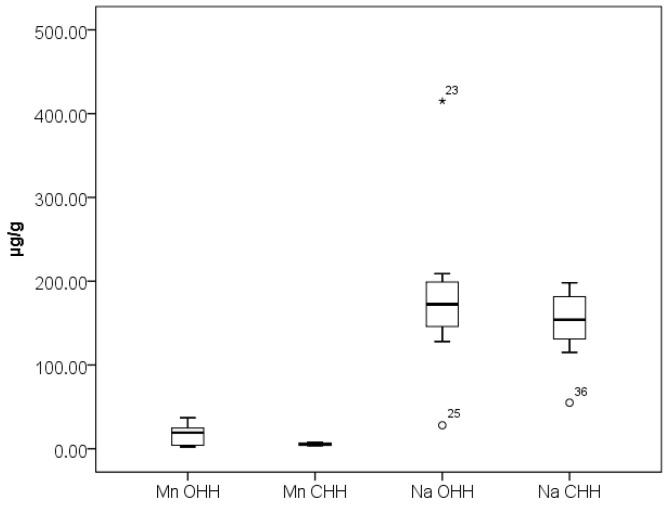
Box plot diagram of contents of Na and Mn in Bulgarian OHH (oak honeydew honey) and CHH (coniferous honeydew honey) expressed as µg/g. Minimal, maximal, and median values are presented. Legend of outliers: OHH 23 (Na)—Tsarevo 2 (410 µg/g), OHH 25 (Na)—Sinemorets 1 (50 µg/g), CHH 36 (Na)—Momchilovsti village (55 µg/g).

**Figure 4:**
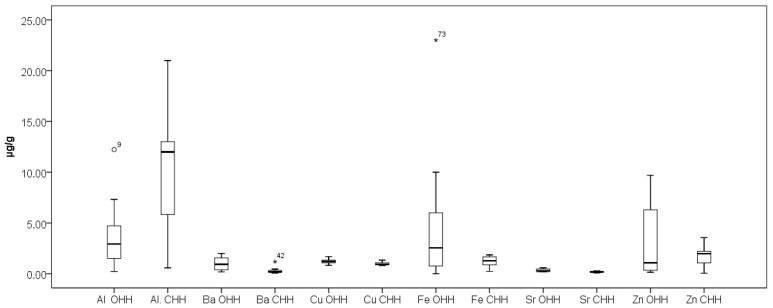
Box plot diagram of contents of Al, Ba, Cu, Fe, Sr, and Zn in Bulgarian OHH (oak honeydew honey) and CHH (coniferous honeydew honey) expressed as µg/g. Minimal, maximal, and median values are presented. Legend of outliers: OHH 9 (Al)—Strandzha Mountain 1 (12 µg/g), CHH 42 (Ba)—Smolyan Lakes 2 (1.19 µg/g), OHH 73 (Fe)—Malko Tarnovo 2 (23 µg/g).

Figure 3 presents the results obtained for elements Na and Mn while Figure 4 presents the results obtained for elements Al, Ba, Cu, Fe, Sr, and Zn in the analyzed OHH and CHH samples. As can be seen from Figure 4, elements Cu and Sr stand out with relatively narrow concentration ranges of their content in OHH and CHH—0.80–1.34 µg/g Cu and 0.090–0.38 µg/g Sr in OHH, and 0.83–1.69 µg/g Cu and 0.18–0.60 µg/g Sr in CHH. Our results are in agreement with the research of Mara et al. [21].

**Figure 5:**
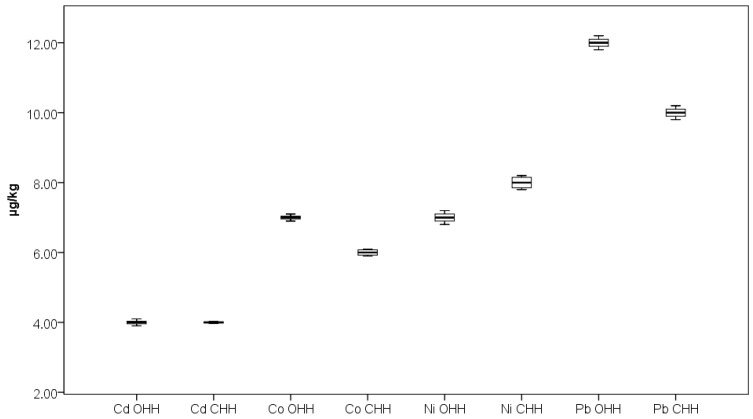
Box plot diagram of contents of Cd, Co, Ni, and Pb in Bulgarian OHH (oak honeydew honey) and CHH (coniferous honeydew honey) expressed as µg/kg. Minimal, maximal, and median values are presented.

As can be seen from Figure 2, Figure 3 and Figure 4, the outlier samples are occasional and not repeated, except for one sample which stands out with higher contents of Ba and Mg in one sample of CHH. These occasional outlier standings are related to different samples, which means that outlying is specifically for concrete samples and no correlation for element content is observed. As can be seen from Figure 5, Co presents in trace levels in the analyzed honey samples. Its content ranges are 7.0 ± 0.1 µg/kg in OHH and 6.0 ± 0.1 µg/kg in CHH.

It is well known that the contents of potentially toxic elements such as Cd, Ni, and Pb in honey are extremely influenced by the area where the beekeeping process is located [43,44]. Ćirić et al. reported the mean contents of Cd, Ni, and Pb to be 3.93, 0.04, and 18.93 µg/kg, respectively, in honey samples from an area with significant industrial activity in Serbia, while the elements’ contents were 1.15, 0.08, and 5.73 µg/kg, respectively, in samples from a predominantly wild Serbian area [43]. Gajek et al. reported mean values of 82 µg/kg for Cd, 177 µg/kg for Ni, and 195 µg/kg for Pb in honey samples from primarily agricultural regions in Poland [44]. Samples from OHH and CHH in our study were collected from predominantly mountainous regions (Figure 1). The results obtained for these potentially toxic elements in Bulgarian OHH and CHH are presented in Figure 5. The contents of elements Cd, Ni, and Pb were found to be 4.0 ± 0.1, 8.0 ± 0.2, and 10.0 ± 0.2 µg/kg in OHH and 4.0 ± 0.1, 7.0 ± 0.2, and 12.0 ± 0.2 µg/kg in CHH. At present, in European Union countries there are no available maximum permissible values for Cd and Ni contents in honey as food. The European Commission has set a maximum level for metals and other elements in honey only for Pb content to be 100 µg/kg [45]. The results obtained for Pb content in our investigation show much lower values than the permitted value which suggests the good quality and safety of the analyzed honey samples.

### 3.3. Correlations

In order to assess the potential correlation between different parameters, Pearson’s correlation coefficients (r) are calculated and data are presented in Table 3. A strong positive linear correlation was found between the color and electrical conductivity of OHH and CHH samples (r = 0.811, *p* < 0.05). Furthermore, honey color is a parameter that can also correlate with other indicators, such as total acidity or total phenolic content [46]. Strong positive linear correlations were found also for elements Ba–Mg, Ba–Mn, Mg–Mn, and Ba–Sr both for OHH and CHH. It is assumed that the elements for which a positive linear correlation was found in the two analyzed types of honey samples (Ba, Mg, Mn, Sr) are not of anthropogenic origin. Their source is probably the soil in which the respective plants were grown. It is likely that the correlating elements have similar behavior on their biological pathway from the soil, through the root system and stem of the plant, to the secreted juices. According to some authors [47,48], elements such as Ba and Sr are commented on as descriptors, with the help of which the geographical origin of various foods could be determined. The results obtained by us can be considered as confirmation of this theory.

## 4. Conclusions

This comparative study successfully demonstrates that Bulgarian oak honeydew honey has physicochemical and elemental profiles that clearly differentiate it from coniferous honeydew honey. The significantly higher electrical conductivity, total acidity, enzyme activity, specific optical rotation, and concentrations of key mineral elements (K, Fe, Zn) in OHH are direct biomarkers of oak honeydew’s origin. These parameters provide a scientific basis for the authentication and quality control of this unique Bulgarian product. The lower HMF in all samples suggests a need to highlight the excellent handling practices of the beekeepers, indicating minimal thermal exposure and optimal storage, which preserves the natural enzymes and freshness of the honey. This work constitutes the first report on the physicochemical parameters and chemical elements of Bulgarian coniferous honeydew honey. Strong positive linear correlations were found for elements Ba–Mg, Ba–Mn, Mg–Mn, and Ba–Sr for OHH and for CHH. Further studies are needed for a more comprehensive characterization, including polyphenolic profiling and bioactivity assays, to fully elucidate its nutritional potential.

## Figures and Tables

**Table 1:** Plant families which were found in analyzed oak honeydew honey (OHH) and coniferous honeydew honey (CHH) samples.

Pollen	OHH	CHH
Main pollens	*Quercus* spp.	*Pinaceae*
Secondary pollens	*Asteraceae*, *Apiaceae*, *Brassicaceae*, *Fabaceae*, *Rosaceae*	*Poaceae*, *Tiliceae*, *Plantaginaceae*, *Fabaceae*, *Lamiaceae*, *Asteraceae*

**Table 2:** Physicochemical parameters of oak honeydew honey (OHH) and coniferous honeydew honey (CHH).

Parameter	OHH Mean ± SD (*n* = 10)	CHH Mean ± SD (*n* = 7)	Significance
Color [mm Pfund]	105 ± 16	63 ± 18	*p* < 0.01
Water content [%]	16.46 ± 1.13	17.51 ± 1.01	NS
Electrical conductivity [mS/cm]	1.01 ± 0.16	0.92 ± 0.17	NS
$\mathrm{Specific}\;\mathrm{rotation},\;{\left[\alpha \right]}_{D}^{20}$	12.5 ± 5.5	−14.32 ± 1.93	*p* < 0.001
pH	4.26 ± 0.20	3.59 ± 0.13	*p* < 0.05
Diastase, Gothe units	22.7 ± 3.3	24.88 ± 1.02	NS
Total acidity, meq/kg	34.0 ± 2.2	33.1 ± 5.6	NS
Invertase, U/kg	121 ± 43	100 ± 28	NS
Proline, mg/kg	287 ± 111	468 ± 145	*p* < 0.001
HMF, mg/kg	6.28 ± 2.08	2.83 ± 0.87	*p* < 0.05

mean ± SD = average ± standard deviation; NS = not significant.

**Table 3:** Calculated Pearson’s correlation coefficients (r) with statistical significance at *p* < 0.05.

Correlation	Oak Honeydew Honey	Coniferous Honeydew Honey
color—electrical conductivity	r = 0.794; *p* < 0.05	r = 0.811; *p* < 0.05
Ba–Mg	r = 0.832; *p* < 0.01	r = 0.783; *p* < 0.01
Ba–Mn	r = 0.890; *p* < 0.01	r = 0.816; *p* < 0.01
Mg–Mn	r = 0.954; *p* < 0.01	r = 0.987, *p* < 0.01
Ba–Sr	r = 0.828, *p* < 0.01	r = 0.818; *p* < 0.01

## Data Availability

The original contributions presented in the study are included in the article/Supplementary Material, further inquiries can be directed to the corresponding author.

## Supplementary Materials

The following supporting information can be downloaded at https://www.mdpi.com/article/10.3390/foods14244298/s1. Table S1. Temperature program for Cd, Ni, Pb determination by ETAAS.

## Abbreviations

The following abbreviations are used in this manuscript:

OHH	Oak honeydew honey
CHH	Coniferous honeydew honey
HMF	Hydroxymethylfurfural
FAAS	Flame Atomic Absorption Spectrometry
FAES	Flame Atomic Emission Spectrometry
ETAAS	Electrothermal Atomic Absorption Spectrometry
ICP-OES	Inductively Coupled Plasma Optical Emission Spectrometry
