# Supplementary material for: Preliminary Characterization of Bulgarian Forest Honeys: Oak Honeydew and Coniferous Varieties
Source: Foods. 2025 Dec 14;14(24):4298. doi: 10.3390/foods14244298 (PMC12733115; doi:10.3390/foods14244298)
Supplement: Supplementary file 1 [file foods-14-04298-s001.zip › foods-3995172-supplementary.pdf]

Table S1. Temperature program for Cd, Ni, Pb determination by ETAAS.

Step	Cd	Ni	Pb
Drying			
Temperature [°C]	120	120	120
Ramp time [sec]	10	10	10
Hold time [sec]	10	10	10
Ar flow [mL/min]	300	300	300
Ashing			
Temperature [°C]	600	1000	600
Ramp time [sec]	10	15	10
Hold time [sec]	20	20	20
Ar flow [mL/min]	300	300	300
Atomization			
Temperature [°C]	1300	2000	1500
Ramp time [sec]	0	0	0
Hold time [sec]	3	5	3
Ar flow [mL/min]	0	0	0
Cleaning			
Temperature [°C]	2400	2400	2400
Ramp time [sec]	2	2	2
Hold time [sec]	3	3	3
Ar flow [mL/min]	300	300	300
